# Recent Progress and Recommendations on Celiac Disease From the Working Group on Prolamin Analysis and Toxicity

**DOI:** 10.3389/fnut.2020.00029

**Published:** 2020-03-17

**Authors:** Katharina A. Scherf, Carlo Catassi, Fernando Chirdo, Paul J. Ciclitira, Conleth Feighery, Carmen Gianfrani, Frits Koning, Knut E. A. Lundin, Detlef Schuppan, Marinus J. M. Smulders, Olivier Tranquet, Riccardo Troncone, Peter Koehler

**Affiliations:** ^1^Department of Bioactive and Functional Food Chemistry, Institute of Applied Biosciences, Karlsruhe Institute of Technology (KIT), Karlsruhe, Germany; ^2^Department of Pediatrics, Polytechnic University of Marche, Ancona, Italy; ^3^Instituto de Estudios Inmunologicos y Fisiopatologicos- IIFP (UNLP-CONICET), Universidad Nacional de La Plata, La Plata, Argentina; ^4^Norwich Medical School, University of East Anglia, Norwich, United Kingdom; ^5^St. James's Hospital, University of Dublin, Dublin, Ireland; ^6^Institute of Biochemistry and Cell Biology, Italian National Council of Research, Naples, Italy; ^7^Leiden University Medical Centre, Leiden, Netherlands; ^8^Department of Gastroenterology, Oslo University Hospital Rikshospitalet and Stiftelsen KG Jebsen Coeliac Disease Research Centre, University of Oslo, Oslo, Norway; ^9^Institute for Translational Medicine, University Medical Center of the Johannes Gutenberg University Mainz, Mainz, Germany; ^10^Plant Breeding, Wageningen University & Research, Wageningen, Netherlands; ^11^INRAE, UR BIA, Nantes, France; ^12^European Laboratory for the Investigation of Food Induced Diseases (ELFID), Department of Medical Translational Sciences, University Federico II, Naples, Italy; ^13^biotask AG, Esslingen, Germany

**Keywords:** barley, celiac disease, gluten, gluten-free diet, Prolamin Working Group, rye, wheat

## Abstract

Celiac disease (CD) affects a growing number of individuals worldwide. To elucidate the causes for this increase, future multidisciplinary collaboration is key to understanding the interactions between immunoreactive components in gluten-containing cereals and the human gastrointestinal tract and immune system and to devise strategies for CD prevention and treatment beyond the gluten-free diet. During the last meetings, the Working Group on Prolamin Analysis and Toxicity (Prolamin Working Group, PWG) discussed recent progress in the field together with key stakeholders from celiac disease societies, academia, industry and regulatory bodies. Based on the current state of knowledge, this perspective from the PWG members provides recommendations regarding clinical, analytical and legal aspects of CD. The selected key topics that require future multidisciplinary collaborative efforts in the clinical field are to collect robust data on the increasing prevalence of CD, to evaluate what is special about gluten-specific T cells, to study their kinetics and transcriptomics and to put some attention to the identification of the environmental agents that facilitate the breaking of tolerance to gluten. In the field of gluten analysis, the key topics are the precise assessment of gluten immunoreactive components in wheat, rye and barley to understand how these are affected by genetic and environmental factors, the comparison of different methods for compliance monitoring of gluten-free products and the development of improved reference materials for gluten analysis.

## Introduction

About 60% of agricultural land worldwide is used to grow cereals, with maize (1,135 × 10^6^ metric tons), wheat (772 × 10^6^ metric tons) and paddy rice (770 × 10^6^ metric tons) as major crops in terms of global production (data from 2017, FAOSTAT^1^). As an important source of dietary nutrients such as carbohydrates, proteins, dietary fiber, vitamins and minerals, wheat is an essential cornerstone for food security. However, the consumption of products made of wheat, and the closely related cereals rye and barley, may cause adverse reactions such as celiac disease (CD), non-celiac gluten/wheat sensitivity (NCGS) and wheat allergy. With increasing evidence from epidemiological studies pointing to a large number of affected individuals in many countries around the world, there is a strong need to understand the fundamental interactions between immunoreactive components in gluten-containing cereals and the human gastrointestinal tract and immune system in order to develop strategies for disease prevention and treatment beyond the gluten-free diet (GFD). The term “gluten” includes the closely related storage proteins of wheat (gliadins and glutenins), rye (secalins), barley (hordeins), and oats (avenins). The part of gluten soluble in aqueous alcohols has been termed prolamins and the insoluble part glutelins.

CD is defined as a lifelong small intestinal immune enteropathy with autoimmune features caused by ingestion of gluten from wheat, rye and barley in subjects with a dominant and necessary genetic predisposition [human leukocyte antigen (HLA)-DQ2 or -DQ8] (1). The main known environmental factor responsible for CD is the consumption of gluten, but there still needs to be a largely unknown factor as initial trigger of the disease. Certain viruses and bacteria are prime suspects, and the idea is that virus infection can prime the immune system in susceptible individuals so that not only the virus is recognized and defeated but the intestinal immune system also misinterprets gluten as “dangerous” (*vide infra*). Patients develop characteristic mucosal (usually IgA) antibodies to the autoantigen tissue transglutaminase [TG2, (2)]. TG2 can deamidate gluten peptides, which improves their presentation by HLA-DQ2 or -DQ8 on antigen-presenting cells of the intestinal mucosa, and this increases their T-cell stimulatory potential (3, 4). While such gluten-specific T-cell responses are characteristic for CD, it is unclear which events cause the loss of mucosal tolerance to food antigens in CD. Many studies now imply a role for additional environmental agents, including the exposure to (intestinal) viruses and bacteria. CD is a systemic disorder that predominantly manifests itself in the mucosa of the upper small intestine (duodenum, proximal jejunum) and is characterized by villous atrophy and crypt hyperplasia, which can vary from mild partial damage to a total absence of villi. As a clinical chameleon (5), CD presents in symptomatic, asymptomatic, potential and refractory forms and can occur at any age. Notably, CD often also presents with a wide variety of extra-intestinal symptoms, including associated autoimmune diseases (6–9). The only effective treatment so far is a GFD that essentially relies on the consumption of naturally gluten-free foods such as animal-based products, fruits, vegetables, legumes and nuts as well as dietary gluten-free products that may not contain more than 20 mg/kg of gluten according to Codex Alimentarius (Codex Standard 118-1979^2^). There are several ongoing attempts to develop non-dietary treatments of the disease—this is briefly discussed later.

Founded in 1985 by Professor Wim Hekkens, University of Leiden, The Netherlands, the Working Group on Prolamin Analysis and Toxicity (Prolamin Working Group, PWG) coordinates multidisciplinary research efforts primarily related to CD. The PWG currently has 13 executive members all of whom are renowned experts in the fields of pediatric and adult gastroenterology, immunology, biochemistry, plant science, food chemistry, and gluten analysis. Building upon this unique multidisciplinary knowledgebase, the PWG has made important achievements both in clinical research into CD and in improving food safety for CD patients by advancing analytical methods for gluten detection.

Some of the highlighted clinical research work of the PWG include the assessment of the safety of oats in the GFD (10–12), the establishment of 10 mg of gluten intake per day as the safe gluten threshold for the vast majority of CD patients (13), the search for wheat species with a reduced content of immunogenic sequences for disease prevention (14, 15), and the study of the signals for T- and B-cell recruitment into the *lamina propria* and epithelial compartment (16).

Having been granted observer status at Codex Alimentarius in 1999, the PWG plays a leading role in the development of enzyme-linked immunosorbent assays (ELISA) for gluten analysis (17) and the validation of such methods in collaboration with the Cereals & Grains Association [formerly known as AACC International; (18–20)], and AOAC International (21). It also produced the only well-characterized reference material, the so-called PWG-gliadin (22) that is used to calibrate a variety of gluten analytical methods and is available in 100 mg batches from the Association of Cereal Research (Arbeitsgemeinschaft Getreideforschung e.V., Detmold, Germany).

During its annual meetings, the PWG regularly unites a select group of about 60 international stakeholders including researchers, celiac disease societies, regulatory bodies, manufacturers of gluten-free foods and raw materials, and manufacturers of test systems for gluten analysis in foods. This paper will report the recent progress and recommendations that were presented and discussed during the last PWG meetings.

## Update on Clinical Aspects of CD

### The Epidemiology of CD

In several countries the epidemiology of CD has been intensively investigated during these last decades (23, 24). In these studies, the incidence of CD is calculated by counting the number of new CD diagnoses in a population over a given period of time, usually 1 year. On the other hand, the overall prevalence of CD is determined through mass CD screening of general population samples. The screening algorithm usually consists of serological tests like IgA class anti-transglutaminase (TG2) antibodies. In some of the studies, positive serology is backed up by gastroscopy with duodenal biopsies for final confirmation of CD on an individual basis. Taken together, these studies have shown that there have been substantial increases in prevalence and incidence over the last two decades (24).

#### Prevalence of CD on a Worldwide Basis

According to a recent meta-analysis, the pooled worldwide prevalence of CD autoimmunity is 1.4% (95% confidence interval, CI: 1.1–1.7%), based on positive results from tests for IgA anti-TG2 and/or anti-endomysial antibodies (so-called seroprevalence). This study found that the pooled global prevalence of biopsy-confirmed CD is 0.7% (95% CI: 0.5–0.9%) with wide regional variations. CD prevalence is 0.4% in South America, 0.5% in Africa and North America, 0.6% in Asia, and 0.8% in Europe and Oceania; it is higher in female vs. male individuals (0.6 vs. 0.4%; *p* < 0.001), and significantly greater in children than adults (0.9 vs. 0.5%) (25). It should however be noted that including only biopsy-confirmed CD cases tends to underestimate the true CD prevalence (as it seems to be the case for North America) since cases of potential CD (CD serology positive with normal/nearly normal intestinal mucosa at the small intestinal biopsy) are excluded from the prevalence calculation. In some European countries, e.g., Sweden, Finland, and Italy, data indeed show a significantly higher overall CD prevalence (1.6–2.3%) (26, 27). Generally speaking, the prevalence of CD is directly related to the population prevalence of HLA-DQ2 or -DQ8 (30–40% in most Western countries) and to the average level of wheat consumed per capita, as shown by data from India: CD is much more common in the Northern part of the country where wheat is the staple food (CD prevalence = 1.2), than in the Southern part with both a lower prevalence of HLA-DQ2/DQ8 and a lower wheat consumption (CD prevalence = 0.13%) (28).

#### The Concept of the Celiac Iceberg

CD screening studies have clearly shown that the percentage of cases that are diagnosed clinically (the visible part of the iceberg) is much smaller than the overall CD prevalence (the submerged CD iceberg). The clinical severity of those detected in regular clinical care and those detected by screening, does not, however, seem to differ (29). In countries showing a high level of awareness of the CD clinical spectrum, still 50–75% of cases remain undiagnosed and are therefore exposed to the risks of long-term complications. In some countries, such as India and China, the visible CD iceberg is <5% of the overall “mountain of ice.” How to increase the CD diagnostic rate (e.g., via mass-screening or case-finding) is still a matter of debate (30, 31).

#### Is CD Prevalence Increasing Over Time?

Studies from several countries, particularly the US, Finland and Italy, suggest that the overall CD prevalence is increasing over time. For instance, the analysis of “old” sera samples taken at two different time-points (15 years apart), coupled with recent population screening data suggested that CD prevalence increased 5-fold in the US during a 50-year period beginning from 1948 to 1954 (32). The environmental factor/s responsible for this huge increase are still unclear (33). A recent study in Denmark showed that the prevalence of diagnosed CD has doubled every decade from 1986 to 2016, the female/male ratio has increased, and also the prevalence of autoimmune comorbidity in 2016 was three times higher among CD patients compared with the general Danish population (34).

### Risk Factors

A number of prospective studies have been performed to identify risk factors for CD. They were focused on the genetic factors predisposing to the disease (in this context the dose of HLA-DQ2 seems to play the most important role) and on environmental factors that increase the risk of developing celiac autoimmunity and then mucosal damage.

#### Infant Feeding

Amongst those studies, two (PreventCD and CeliPrev) have carried out an intervention based on the timing of gluten introduction in infants. Other observational studies have assessed the relationship between infant feeding practices and the risk of CD (Generation R, Norwegian Mother and Child Cohort Study, BabiDiab, TEDDY). In general, prospective studies have not been able to confirm the previous findings that both age of gluten introduction and breastfeeding influence CD risk (35, 36). Recent epidemiologic studies reported a positive correlation between the incidence of CD cases and the amount of gluten in the diet within the early years of life (37–39), but there are also reports questioning this relationship (40–42). Further immunological and multicentre studies are mandatory to assess whether a reduced gluten exposure in early life may protect from CD onset in predisposed individuals.

#### Early Events

A large registry-based cohort study that included over 1.5 million children from Denmark and Norway found no association between the mode of delivery (cesarean section vs. vaginal birth) and the risk of diagnosed CD (43). Data collected from the same cohort indicated that exposure to systemic antibiotics in the first year of life was positively associated with diagnosed CD, with a dose-dependent relation between an increasing number of dispensed antibiotics and CD risk (44). However, a recent systematic review of two studies on prenatal and three studies on postnatal antibiotic exposure reported contradictory results and thus rather excluded an association between antibiotic use and the risk of developing CD (45), as already suggested by the TEDDY study (46).

#### Infections

Longitudinal prospective studies have suggested an association between frequent rotavirus infection and an increased risk of CD (47). A protective effect of rotavirus vaccination has also been reported (48). Both reovirus and norovirus (49) have been shown to be able to break oral tolerance in murine models and there is evidence for the role of reovirus in the pathogenesis of CD (50). In addition, infections with enterovirus A and B, especially with high titer and long duration, during early childhood were associated with later CD, whereas adenovirus infections were unlikely to contribute to CD onset (51). Interestingly, also the occurrence of acute respiratory infections seems to play a role (52).

#### Microbiota

Microbiota has been hypothesized to influence the risk of developing CD. Studies on active CD patients have suggested that microbiota from CD patients may harbor more pathogenic or proinflammatory bacteria (53, 54). However, in such studies on active CD patients, it cannot be stated whether dysbiosis is a risk factor for CD or a consequence of mucosal damage and inflammation. In infants carrying the high risk genotype a reduced number of Bifidobacterium (*B. longus*) was found. Early alterations of the proportions of Firmicutes were noted in children who later progressed to CD (55). However, in another study no statistically significant differences in the fecal microbiota composition were found between children who later developed CD and the control children without disease or associated autoantibodies (56). Mouse experimental studies, including fecal transplants from patients, demonstrated a protective effect of certain lactobacilli that are able to degrade immunogenic gluten peptides, thus alleviating small intestinal damage (57). Microbe-host interactions were recently identified as relevant factors in the development of food sensitivities. Duodenal biopsies from CD patients displayed increased proteolytic activity due to higher abundance of Proteobacteria that express gluten-degrading enzymes such as elastase (LasB). This resulted in the activation of an elastase-dependent, but gluten-independent inflammatory response mediated by the protease-activated receptor 2 pathway. In the presence of CD risk genes, a synergistic effect between elastase and gluten was demonstrated, thus highlighting the importance of microbiota in modulating the host immune response (58).

### Natural History

The natural history of CD proceeds based on the prominent genetic risk (HLA-DQ2/DQ8) and exposure to environmental risk factors finally to break the oral tolerance to gluten. The seroconversion with the appearance of anti-TG2 autoantibodies (CD autoimmunity) is considered to be a sign of activation of anti-gluten adaptive immunity, being sustained by gluten-specific T cells. However, not all subjects in this stage appear to progress further to villous atrophy and consequent malabsorption (59). Some may remain at this stage with no histological damage or very mild lesions. This condition has been dubbed “potential CD.” In a subset, anti-TG2 antibodies may fluctuate or even disappear (60, 61). Recently, factors predicting such evolution have been suggested, such as increased density of intraepithelial γδ T cells, small intestinal mucosal deposits of anti-TG2 antibodies, and HLA dose (62).

### Clinical Gluten Challenge and the Adaptive Response to Gluten

For decades, the roles of the adaptive and the innate immune system as the key players in CD immunopathogenesis have been discussed. The very clear genetic association primarily to HLA-DQ2.5 and to a lesser degree to HLA-DQ2.2 and HLA-DQ8, and the finding that these HLA molecules present gluten to *lamina propria* CD4^+^ T cells all argue for a prominent role of the adaptive immune system (63–66). In addition, there is a very strong HLA-DQ2 gene dose effect which correlates with stronger gluten-specific T-cell responses in individuals homozygous for HLA-DQ2.5 compared to heterozygotes (67). Very recent studies demonstrated that the DQ2.5 genes are more expressed than non-CD associated alleles in antigen-presenting cells heterozygous for DQ2.5. This differential expression of CD risk genes affects the level of the encoded DQ2.5 molecules on the cell surface and the strength of gluten-specific CD4^+^ T-cell response (68–70). According to these findings, the magnitude of the T-cell response appears more prominent dependent on the amount of gluten and less on the DQ2.5 gene doses. Although innate effects of gluten also may be important, these are generally only found in patients with CD and not in healthy individuals. *Lamina propria* CD4^+^ T cells recognize certain peptides from the gluten protein types α/β-, γ-, ω-gliadins, and high-molecular-weight glutenin subunits (HMW-GS) where the common denominator of this peptide recognition is that the peptides are deamidated by the enzyme TG2 (2, 3, 71, 72), although some sequences do not need deamidation to be CD-active. The set of peptides presented by any given of the two CD-associated HLA molecules share common features where the charged amino acids of the gluten peptides fit into pockets of HLA-DQ2/DQ8 molecules (9, 73–75). The CD4^+^ T cells preferentially recognize these, partly deamidated peptides that cluster in proline- and glutamine-rich stretches of the gluten proteins. However, the peptide sequence of only ≈50% of the total number of such *lamina propria* T cells can be accounted for. Although often termed an “autoimmune” disease, this is mainly related to the production of autoantibodies to TG2, as hallmark of CD, while T cells recognizing TG2 do not appear to play a role.

The gluten-specific T cells can be demonstrated by *in vitro* culture of biopsies from CD patients, as first shown in the early 1990's (64, 65). They can also be demonstrated by direct staining using so-called HLA-DQ:gluten peptide tetramers; i.e., tetramers of HLA-DQ molecules with gluten peptides bound (76). It was shown that a short gluten challenge will mobilize gluten-specific T cells into the peripheral blood (77, 78), and such cells can be quantified by ELISpot or by HLA-DQ:gluten tetramers (79). Such T cells express markers for gut-homing, but if they actually are mobilized from the intestine remains uncertain. The procedure can be used for diagnostic purposes after gluten challenge (80) and may perform better than a 2-week gluten challenge followed by upper endoscopy with biopsy (81). Furthermore, employing an improved methodology, such HLA-DQ:gluten tetramer^+^ cells can be detected without gluten challenge and clearly distinguish CD patients and healthy individuals (82, 83). When the HLA tetramer technology was coupled with the CyTof technology, it was found that these cells carry a surprisingly rare phenotype with a profile suggesting that they may help plasma cells. Importantly, they are similar in profile to disease-relevant T cells in other autoimmune diseases, where the antigen specificity is unknown (84). Most recently, Zühlke et al. have demonstrated that the HLA-DQ:gluten tetramer^+^ cells after gluten challenge show interesting features: (1) the kinetics of appearance peaks between day six and eight, (2) there are large inter-individual differences in numbers of cells, (3) even a one-day, single challenge with gluten mobilizes detectable cells, (4) although the numbers of cells vary between individuals, expression of the activation marker CD38 on the HLA-DQ:gluten tetramer^+^ cells is a very specific and sensitive parameter (85). Thus, the HLA-DQ:gluten tetramers may be developed as a powerful diagnostic tool for CD but are not yet available outside the research setting and are not approved by any guidelines (86).

The importance of the adaptive immune system has recently been strongly supported by finding of the *bona fide* T cell cytokine IL-2 as fast as 4 h after ingestion of gluten. This was first shown after intradermal injection of gluten peptides but the same is seen after peroral gluten exposure (87). No such response is seen after gluten intake by non-celiac, gluten-free subjects (88). It is conceivable that either the HLA-DQ:gluten tetramers or the IL-2 response can be used as surrogate markers for testing of therapies for CD; this is the focus of ongoing research.

### Compliance With the Gluten-Free Diet

Although a strict GFD remains the only effective treatment for CD, the rate of compliance is far from 100%. Adherence to the diet is higher in children and, in general, in those who have received diagnosis in early childhood (89). Socioeconomic factors, sex, access to health care facilities also influence the level of compliance. Adherence to the GFD is not easy to assess, clinical improvement not being a valid criterion. Periodic interviews conducted by dieticians could monitor compliance, with structured short, validated, dietary questionnaires being an alternative to consultations with a dietician (90). Anti-TG2 serology is in clinical practice the most used current method. In fact, antibody titers decrease after a few weeks on a strict GFD, but sometimes it can take longer, particularly if high titers are present at diagnosis. In any case antibody measurement cannot reveal minor dietary transgressions (91). The best way remains performing duodenal biopsies, but this is invasive and should be reserved to cases with no clinical improvement or no decrease of serological titers. More recently detection of gluten immunogenic peptides (GIP) in feces and urine has been proposed as new biomarker to detect gluten intake and verify GFD compliance in CD patients. Their determination is non-invasive and relatively simple, but shows poor correlation with antibody levels or with the response to dietary questionnaires (92).

## Update on Gluten Digestibility and Development of Non-Dietary Treatment

### Gluten Digestibility Influences Its Stimulatory Properties

Gluten, the trigger factor of CD, is composed of hundreds of monomeric, oligomeric, and polymeric proteins, these latter interlinked by disulfide bonds (93). The unique amino acid composition of gluten proteins, enriched in glutamine and proline residues, makes this important dietary component highly resistant to gastrointestinal digestion (94). The inability of gastric and pancreatic proteases, as well as of the brush border membrane endopeptidases, to cleave proline-glutamine bonds throughout the gluten protein sequences leads to peptide fragments of different lengths that escape proteolytic degradation (95). These gluten peptides retain a marked immunogenic potential, as they pass across the small intestinal epithelial barrier and may trigger an adverse immune response in genetically susceptible individuals (96). It has been demonstrated that in patients with CD some long gluten peptides are site-specifically deamidated by TG2, bound to HLA class II molecules of antigen-presenting cells, and stimulate a specific immune response mediated by CD4^+^ T cells. These mucosal T cells proliferate and release several inflammatory cytokines, such as interferon-γ and interleukin-21 with a key role in activating the injurious process of villous atrophy (97). Recently, the nomenclature of the existing CD-relevant gluten epitopes recognized by CD4^+^ T cells has been updated (98). However, it has to be emphasized that the pool of CD-active sequences is far from being complete to date as the epitopes recognized by many T cells are not known. The clinical importance of these sequences is, however, uncertain.

A “new” class of poorly digestible proteins in wheat, the amylase/trypsin-inhibitors (ATI), has received a lot of attention recently, but their potential role in the pathogenesis of CD needs further investigation (99–101).

### Gluten Degradation as a Treatment

The current therapy for CD patients is the lifelong withdrawal of gluten from the diet. Although, the GFD is efficacious in the great majority of patients, with the restoration of mucosa villous morphology and function, many young patients are poorly compliant, so that the identification of an alternative treatment would be beneficial for those patients for whom the GFD fails or is impracticable (102). Recently, great efforts were made to identify a pharmacological therapy that could be used to replace or support the GFD for treatment of CD patients (103). Currently, several proteolytic enzymes of microbial or plant origins have demonstrated a high efficiency to quickly degrade gluten proteins at very low pH, as occurring in gastric conditions (104–107). These glutenases, thanks to their efficacy in cleaving the proline- and glutamine-rich gluten sequences are promising drugs to abolish the immunogenic potential of dietary gluten. Both *in vitro* and pre-clinical studies have shown that the glutenase treatment results in a marked reduction of the amount of gluten epitopes in wheat-containing food. The possibility of preventing that gluten immunogenic peptides reach the duodenal mucosa strongly suggests the possible use of glutenases in oral enzymatic treatment for CD (108). AN-PEP, a prolyl endopeptidase from *Aspergillus niger*, even though it was not intended to replace a GFD, was effective as a digestive aid protecting against the unintentional intake of gluten (109), or when consuming food which may contain small amounts of gluten, e.g., beer. A recent study demonstrated that the endopeptidase E40 from *Actinoallomurus* A8 is a fast-acting and strongly efficient glutenase, and thus a candidate as enzyme adjuvant to a GFD for the dietary management of CD (104). Glutenases can also be induced in wheat by germination but the activity is not high enough to be useful as an oral food supplement. However, this strategy can, for example, be used to eliminate residual gluten from food such as beer (110). Special wheat lines were developed recently to express the barley endoprotease B2 combined with a prolyl endopeptidase from *Flavobacterium meningosepticum* or *Pyrococcus furiosus* that significantly reduced the amount of indigestible gluten peptides (111). Sequence guided site-saturation mutagenesis was used to enhance the thermostability of these enzymes and allow their use in heat-treated cereal products (112).

### Development of Other Non-dietary Treatment Options

As repeatedly stated in this paper, the GFD is a well-established and effective treatment for CD, at least as long as the patient is fully compliant. Here it is also important to emphasize that a GFD is inherently associated with nutritional deficiencies and not recommended except in the treatment of gluten-related disorders (113). It may be noted that no randomized, controlled trials have been performed to prove the real effects of the treatment, but this is not unusual in medicine. CD patients themselves express huge interest in non-dietary treatments like drugs or vaccines (114). Attractive options include sequestering of gluten within the lumen, luminal digestion of gluten by exogenous enzymes, interfering with mucosal integrity (tight junctions), inhibition of TG2, inhibition of antigen presentation, immune skewing and re-establishment of oral tolerance or clonal deletion, to mention a few (115–119). A plethora of Phase 1 studies, a small handful of Phase 2 and a single Phase 3 study is ongoing at the moment, but no drugs have reached the market. Almost all of these studies are based on preclinical studies *in vitro* and *ex vivo*. This research is hampered by the lack of good line research opportunity as the immune reaction to gluten is dependent on intact mucosal interaction. It is also hampered by lack of good animal (mouse) models for CD, although there are several mouse models for immune reaction to gluten (120, 121). Recently, a mouse model was developed that reproduces the overexpression of interleukin-15 (IL-15) in the gut epithelium and lamina propria, expresses the predisposing HLA-DQ8 molecule, and develops villous atrophy after ingestion of gluten (122). At any rate, it can be foreseen that such non-dietary options will come to the market, either as add-on therapy to the GFD, as rescue therapy after incidental gluten exposure or as replacement of the GFD.

## Update on Gluten Composition of Wheat and Methods for Modification

### Gluten Content and Composition in Wheat Species and Cultivars

The availability of the first annotated reference sequence for the hexaploid bread wheat genome containing 107,891 high-confidence gene models (123) recently allowed the establishment of a genome reference map for immunostimulatory wheat proteins (124). One of the hypotheses being discussed to explain the increasing prevalence of CD is that the protein composition of wheat may have changed over the past decades due to breeding and agronomic practices. The main goals of wheat breeding are increased yield, improved resistance against plant diseases, pests, and climatic stress, more efficient use of fertilizers as well as increased protein content. The protein content represents one of the key quality aspects that significantly influences the bread wheat quotation worldwide. With CD being determined by gluten as major and necessary environmental risk factor and HLA-DQ2/DQ8 as genetic risk factors, one might envisage to be able to predict the prevalence of CD in different countries. A systematic worldwide compilation of this data revealed that those two factors are clearly required for the development of CD, but not suitable to predict the prevalence. There was no correlation between CD prevalence, the levels of wheat consumption and the frequencies of HLA-DQ2/DQ8 or the combination of both. This rather surprising result was primarily due to several outlier populations in regions such as north-western India, northern Africa, Mexico, Finland, and Russia. For example, the prevalence of CD in Finland is among the highest worldwide (2.4%), whereas that of neighboring Karelia (Russia) is very low (0.2%), although both regions share similar levels of wheat consumption and frequencies of HLA-DQ2/8 (125). Within the United States, CD was 5.4-fold more common among individuals who lived at latitudes of 40° North or more than among individuals who lived at latitudes below 35° North, independent of race or ethnicity, socioeconomic status, and body mass index (126). This discrepancy can only be explained by further environmental factors that cause a loss of tolerance to dietary gluten and initiate CD (23, 24). Although there was no clear trend toward higher protein or gluten contents since the 1950s (127), the selection criteria for breeding might have resulted in a higher immunostimulatory potential of wheat (128). Several studies have explored the protein composition of different wheat species and cultivars of hexaploid bread wheat (*Triticum aestivum* subsp. *aestivum*) and spelt (*T. aestivum* subsp. *spelta*), tetraploid durum wheat (*T. turgidum* subsp. *durum*), and emmer (*T. turgidum* subsp. *dicoccum*) as well as diploid einkorn (*T. monococcum*) with respect to their content of potentially immunostimulatory proteins (15, 129–131).

There is evidence for changes in gluten protein composition (132–134) with decreasing contents of gliadins and total gluten, but increasing contents of glutenins from diploid to tetraploid and hexaploid wheats. However, within bread wheat or durum wheat, there were no clear differences in the contents of selected CD-active epitopes between modern cultivars and landraces not subjected to breeding (135–138). All studies consistently reported a significant effect of environmental conditions on the expression of CD-immunogenic peptides, with e.g., low or high cultivation temperatures affecting the expression of immunostimulatory proteins in different ways (124). This high variability in protein composition regulated by mechanisms in the wheat plant that are still incompletely understood complicates the search for specific cultivars that naturally express low amounts of CD-immunogenic peptides independent of the environmental conditions.

Recent studies have shown that gluten proteins of several einkorn (*T. monococcum*) landraces have a reduced capability of activating the mucosal innate immune cells and inducing enterocyte apoptosis (14). Other studies have attributed the reduced immunotoxicity to the presence of protective sequences (139). A recent study demonstrated that wheat gluten from einkorn is extensively degraded by the gastrointestinal protease cocktail, including endopeptidases of the villous brush border membrane (15). This results in the release of a reduced amount of peptides that can activate pathogenic CD4^+^ T cells in the gut mucosa. Altogether, these findings are relevant from the perspective of disease prevention, taking into account that the incidence of CD is much higher (≈10%) in first-degree relatives of CD patients carrying the high-risk HLA-DQ genes (36). However, the evidence cited in support of *T. monococcum* is still insufficient, and it is not recommended to include this wheat species into the diet of CD patients.

### Removing Gluten From Wheat

It is likely, that no existing wheat species or variety is completely safe for use by CD patients, as they all contain far more than 20 mg/kg of gluten. Can we specifically develop wheat varieties that are CD-safe? In barley, an ultra-low gluten variety has been developed that is safe for CD patients, as it contains <20 mg/kg of gluten. It was developed by combining existing induced recessive mutations through breeding (140). This strategy is very difficult to implement in bread wheat as it has three genomes and one does not want to remove all gluten genes (141), but other approaches may be successful (142, 143). The general applicability of these breeding approaches needs to be discussed, since only gluten endows the dough with the desired properties for bread making.

Using RNA interference (RNAi), several groups have shown that it is possible to strongly reduce the expression of α-gliadins (144), γ-gliadins (145), ω-gliadins (146, 147), or all of them (148) in wheat. In the latter study, the α- and ω-gliadins were downregulated to the extent that no CD epitopes could be detected using LC-MS/MS (148). Unfortunately, these lines are genetically modified (GM) as the RNAi construct must remain present. As no GM wheat has been commercially introduced anywhere in the world, it is unlikely that these lines will reach the market shortly.

A recent alternative technology is to use gene editing with CRISPR/Cas9 to delete gliadin genes in order to produce gluten-free wheat and/or to edit the epitopes in them to generate wheat with safe gluten. Sánchez-León et al. (149) targeted two conserved sites adjacent to the epitope-containing region in the α-gliadin genes. Up to 35 of the 45 α-gliadin genes were mutated in a single line, with small or larger deletions around the target sites. This line showed a 85% reduction of the R5 and G12 ELISA signals. Jouanin et al. (150) simultaneously edited multiple sites in α- and γ-gliadins with a single construct. Although the lines produced in these pilot studies are not yet safe, they demonstrate the power of gene editing for effectively modifying tens of genes of multiple gene families in a polyploid species at once. The Cas9 construct used to generate the edits is removed afterwards by crossing, leaving only mutations that are identical to what can occur naturally. In most of the world, the resulting plants are not considered as GM, with the exception of the EU (151).

## Update on the Use of ELISA for Gluten Analysis

### Advances in Compliance Monitoring of Gluten-Free Products

Apart from evolving proteomics-based detection methods, enzyme-linked immunosorbent assays (ELISAs) are most commonly used to monitor the compliance of gluten-free products to the regulatory threshold of 20 mg/kg of gluten (Codex Standard 118-1979^2^). There are more than 20 ELISA test kits on the market that use different principles (sandwich vs. competitive), extraction procedures, reference materials for calibration and various polyclonal or monoclonal antibodies such as the Skerritt (401.21) (152), R5 (153), G12 (154), and α20 (155). A wide variation in the reported measurement results between different commercial kits were observed in several studies (156–158). These discrepancies were mainly attributed to the use of different reference materials and to the fact that the used antibodies target only a fraction of gluten components whose proportions may vary according to the contamination source. The ELISA R5 Mendez Method is currently laid down as a Codex type I method for gluten determination in foods and, therefore, continues to be the most widely used assay. However, the R5 ELISA has two disadvantages; first it overestimates gluten from rye and barley when calibrated to gliadins and secondly, it does not detect glutelins adequately and the gluten content is calculated by multiplying the prolamin content detected by a factor of two. To address these limitations, a new sandwich ELISA based on four different monoclonal antibodies was developed that detects prolamins from wheat, rye and barley as well as HMW-GS, HMW-secalins from rye and low-molecular-weight (LMW)-GS from wheat. The performance of the test kit was recently validated for the quantitative analysis of wheat, rye and barley gluten in oat and oat products by an international collaborative study with 19 laboratories. The results of the study showed recoveries ranging from 99 to 137% for wheat, rye and barley when analyzing defined validation materials (159, 160) and relative reproducibility standard deviations from 10 to 53% for samples containing 10 mg/kg of gluten or higher. Following review by the AOAC Expert Review Panel for Gluten Assays, the method was adopted as AOAC *Official Method* First Action 2018.15 (21).

### A Five Cultivar Wheat Blend Is Suitable for New Reference Material Production

Reference materials that are representative of the target analyte are essential prerequisites for calibrating and assuring the accuracy of analytical methods. They form the basis for method establishment and validation, proficiency tests, and verification of the comparability between different methods and laboratories (161–163). The use of appropriate reference materials was recently shown to efficiently reduce the disparity of gluten analysis between different commercial kits (157). A variety of different reference materials are used in ELISA test kits for gluten analysis including wheat or gluten preparations, some of which are proprietary to the respective kit manufacturer with little information on their exact composition related to immunoreactive sequences. Food matrix and food processing are also known to influence analytical results, which is why incurred materials are recommended to reflect the properties of actual food samples as closely as possible.

The best characterized reference material available for gluten analysis is the so-called PWG-gliadin that was developed by our group of experts. PWG-gliadin constitutes the purified gliadin fraction extracted from a mixture of the 28 most common European wheat cultivars, as of 1999 (22). It is homogeneous, completely soluble in 60% ethanol, representative for European wheat, regularly monitored for stability and widely used to calibrate ELISA test kits and other methods for gluten analysis (164, 165). However, as its supply is limited, efforts to develop new reference and incurred materials are currently underway. A collection of wheat cultivars from different countries was characterized for gluten protein composition and ELISA response to establish selection criteria and identify cultivars that are as representative as possible for the multitude of cultivars grown worldwide. A blend of the selected five cultivars from Asia (Yumai-34), Australia (Yitpi), Europe (Akteur, Mv Magvas), and North America (Carberry) was further characterized and appears to be suitable for further reference material development (166, 167).

## Codex Alimentarius and Gluten Quantitation by ELISA

Codex analytical methods are being revised every 10 years and revision of the R5 Mendez ELISA was due in 2018. Thus, it is time to discuss how to handle ELISAs for gluten quantitation regarding approval by the Codex Alimentarius. Based on the matrices used for validation, the R5 method has been recommended for gluten quantitation in maize matrices and the G12 method for the analysis of rice matrices. Both methods fulfill the performance requirements for gluten analysis set by the Codex Standard 118-1979^2^, i.e., a limit of quantitation of 10 mg gluten/kg or less and the detection of CD-active epitopes. The recently developed Total Gluten ELISA covers both gluten fractions and, thus, measures the gluten content (21). This is an important step forward compared to the R5 and G12 ELISAs that measure the prolamin content and this is then converted into the gluten content by multiplication with the factor of two.

### Concerns

A major concern, in particular of celiac societies, CD patients, food producers, and national food control laboratories is the unclear situation, if several ELISA methods for gluten quantitation were endorsed. It can be assumed that each analytical laboratory would use one ELISA as the default method for gluten quantitation. Consequently, it would be unclear, if a value obtained by one laboratory with one kit would be comparable to the value provided by a different laboratory with a different kit. This would lead to the question of how to handle conflicting results from different laboratories. In general, the possibility of having two type I methods has to be questioned, because the definition of a type I method as “the only method” should exclude approval of a second type I method. On the other hand, if a proprietary method fulfilling the performance criteria of the Codex is on the market and has been approved by suitable collaborative studies, it should not be excluded due to the fact that another method has already been endorsed by the Codex Alimentarius. Both the R5 and G12 sandwich ELISAs have been compared in a number of scientific studies. In summary, the results of these studies strongly suggest that these methods do not yield comparable results. Typical examples are papers published by Bugyi et al. (156), Bruins Slot et al. (168), and Scherf (158).

### Position of the PWG

Therefore, the PWG suggests that ELISA methods should be approved using a combination of

1. information on the method that has been used to provide the analytical value,
2. strict performance criteria and
3. a pre-defined set of maximum five matrices.

This would be similar to the Standard Method Performance Requirements (SMPR) published by AOAC International for allergen-containing commodities such as whole egg, milk, peanut, and hazelnut (169).

#### Performance Criteria

Performance criteria include the correct setup and statistical evaluation of validation studies (161, 170, 171) as well as the fulfillment of the requirements for standard method performance (172). The minimal performance requirements set in AOAC SMPR 2016.002 (169) for whole egg, milk, peanut and hazelnut can be adapted to gluten. Possible performance criteria are summarized in Table 1. AOAC suggests low recovery rates of 60%, but the PWG feels that, in general, the recovery range should be between 80 and 120 %, which is in line with Abbott et al. (161). With respect to LOD and LOQ, the three ELISAs under consideration perform well and meet the requirements (18–21).

**Table 1:** Method performance requirements for gluten ELISAs.

**Parameter**	**Value/Range**
Analytical range (mg/kg)	5–100
Limit of detection (LOD) (mg/kg)	3
Limit of quantitation (LOQ) (mg/kg)	10
Recovery (%)	80–120
Repeatability relative standard deviation (RSD_r_) (%)	20
Reproducibility relative standard deviation (RSD_R_) (%)	30

#### Matrices

Matrices should not be based on botanical origin (e.g., rice- or maize-based), but on constituents that most likely affect the interaction of the antibodies with the gluten antigens. Possible matrices should be categorized into protein-based, starch-based, fat-based, polyphenol-rich, and fiber-rich foods (173). Table 2 suggests categories and examples for foods from each category. Examples are limited to three per category to keep the number of required analyses in validation studies in a range that can be handled. Kit manufacturers are encouraged to agree on a set of matrices which should be comparatively analyzed using their methods. In case of conflicting R5/G12 results, in particular in the concentration range of the 20 mg/kg threshold, the higher concentration value should be considered relevant in the interest of the celiac consumers. In future analyses, it should then be avoided having to do two ELISAs. For any analysis value, the ELISA method that was used should be indicated alongside the results.

**Table 2:** Suggested matrix categories and examples for foods from each category.

**Category**	**Examples**
Protein-based	Coated meat, sausage, protein isolate/concentrate
Starch-based	Starch, baked goods, sauce
Fat-based	Cookie, cake, ice cream
Polyphenol-rich	Chocolate cake, cocoa powder, beer
Fiber-rich	Cereal bran, breakfast cereals, legume seed flours

## Recommendations for Future Research and Actions

### Clinical Aspects of CD

Based on the most recent findings regarding epidemiological and clinical aspects of CD as discussed above, the PWG recommends the following priority research areas. With the recognition of CD just beginning to emerge, particularly in sub-Saharan Africa and Eastern Asia, more data needs to be collected in order to make a robust estimate of the prevalence of CD in these parts of the world. Recent epidemiological findings from Denmark, Finland, Italy and the US suggest an increasing prevalence of CD over time, but the reasons for this still remain unknown. Among the factors being suggested are genetic and epigenetic as well as environmental factors of which infectious agents are most likely to play a role. In order to assess the specific contributions of these factors toward increasing the risk of CD development, the natural history of the disease needs to be understood in more detail. Currently, there are still gaps in our knowledge on how the disease proceeds from the genetic risk combined with exposure to environmental risk factors in the initial loss of oral tolerance to gluten. Even then, some individuals remain at this stage of activated anti-gluten adaptive immunity with no or very mild histological abnormalities, whereas others progress to full-blown villous atrophy. It will be critical to clarify the role of microbiome/virome changes and infections in the period preceding the development of CD and to find markers (epigenetic changes, genetic expression, metabolome alterations, T-cell markers) that predict the development of the disease at the earliest stage possible. Related to this, it will be equally important to identify the factors controlling evolution from CD autoimmunity to mucosal damage and the biomarkers predictive of such evolution to enable the identification of preventive measures and non-dietary treatments. Finally, despite increased awareness of CD, diagnostic delays are still common and the appropriate policy to be implemented to improve the diagnostic rate needs to be determined.

### Analytical Aspects of Gluten

For reasons of better handling and long-term stability compared to flours as evidenced by the excellent properties of PWG-gliadin since its production almost 20 years ago, we continue to support the use of isolates as reference materials. Because PWG-gliadin only constitutes the alcohol-soluble fraction of wheat gluten, we aim to provide prolamin and total gluten isolates from wheat, rye and barley flours, respectively. The first steps will be to establish a suitable protocol to extract all relevant immunoreactive gluten proteins from the flours, characterize the exact composition of the isolates and ensure homogeneity and solubility. Research efforts to identify representative rye and barley cultivars have just started as well as fundamental studies on suitable extraction protocols. We recommend using the same reference material for calibration of analytical methods for better comparability and reproducibility of results.

The PWG acknowledges that more than one ELISA method for the analysis of gluten in foods are currently used and that the results of these methods are not comparable. The group does not support the policy of the Codex Alimentarius to allow approval of more than one type 1 method, because this is in disagreement to the definition of a type 1 method. The Codex Alimentarius should decide soon how to proceed, because several methods are currently already available that fulfill all performance criteria such as the R5, G12, and Total Gluten ELISAs. The PWG suggests that performance data of these ELISAs obtained with identical or at least comparable matrices should be compared. If existing data is not sufficient, comparative studies need to be carried out on the set of foods suggested in this paper. This could result in a kind of guidebook suggesting specific ELISAs for specific foods.

## Author Contributions

KS, CC, CG, KL, MS, RT, and PK wrote the first draft of the manuscript. FC, CF, FK, DS, and OT contributed to revising and editing the manuscript. All authors read and approved the final manuscript.

### Conflict of Interest

The authors declare that the research was conducted in the absence of any commercial or financial relationships that could be construed as a potential conflict of interest.

## Footnotes

^1^Food and Agriculture Organization of the United Nations (accessed December 11, 2019).

^2^Codex standard for foods for special dietary use for persons intolerant to gluten. Codex Alimentarius Commission; revision 2008, amendment 2015.
